# Intact Parathyroid Hormone Reference Intervals: Importance of Pre-Processing for Indirect Reference Interval Estimation

**DOI:** 10.3390/diagnostics16152305

**Published:** 2026-07-23

**Authors:** Anne Meyer, Robert Müller, Markus Hoffmann, Tobias Fischer, Øyvind Skadberg, Matthias Orth

**Affiliations:** 1 Abbott GmbH, 65205 Wiesbaden, Germany; 2 Department of Medical Biochemistry, Stavanger University Hospital, 4021 Stavanger, Norway; 3 Institute of Laboratory Medicine, Vinzenz von Paul Kliniken gGmbH, 70027 Stuttgart, Germany

**Keywords:** intact parathyroid hormone, iPTH, refence interval, reference range, reference limit, data pre-processing, indirect methods, RefLim, TML, refineR

## Abstract

**Background/Objectives:** This study examined the impact of data pre-processing on indirectly derived reference intervals for intact parathyroid hormone (iPTH). Routine laboratory data from a single Norwegian site, measured on the Abbott Alinity i analyzer, were processed with different pre-processing steps before applying and comparing three indirect estimation methods. **Methods:** Data pre-processing consisted of several steps, including filtering based on the number of results per patient, exclusion of specific hospital wards, and the use of predefined biomarkers. Reference intervals were estimated for datasets subjected to different pre-processing strategies using three indirect methods: RefLim, truncated maximum likelihood (TML), and refineR. The impact of pre-processing on the derived reference intervals was systematically evaluated. In addition, vitamin D status and estimated glomerular filtration rate (eGFR) were evaluated as potential partitioning criteria. For comparison, reference intervals were also calculated using both conventional parametric and nonparametric estimation methods. **Results:** Data pre-processing had a pronounced impact on the estimated reference intervals. Restricting the dataset to subjects with a single measurement proved to be an effective strategy for excluding pathological patients, whereas exclusion of specific hospital wards and biomarker-based filtering had a negligible additional effect. **Conclusions:** While stringent data pre-processing and large initial datasets are essential for reliable indirect reference interval estimation of complex analytes such as iPTH, the choice of pre-processing strategy must be guided by analyte-specific characteristics, as different analytes may require different approaches.

## 1. Introduction

Parathyroid hormone (PTH) plays a central role in regulating plasma calcium (Ca) and phosphate levels, acting in coordination with vitamin D (VitD) and magnesium (Mg) by modulating intestinal absorption and bone resorption. Intact parathyroid hormone (1–84) (iPTH) plays a central role in the pathogenesis of hypo- and hyperparathyroidism and is essential for distinguishing PTH-mediated hypercalcemia from other causes of elevated Ca levels. Primary hyperparathyroidism is caused by autonomous secretion of PTH from the parathyroid glands, irrespective of serum Ca concentrations. Secondary hyperparathyroidism is characterized by a reactive, compensatory increase in PTH secretion resulting from chronic disturbances in Ca, phosphate, and/or VitD homeostasis [1,2]. Accurate reference intervals (RIs) for iPTH are essential for the correct diagnosis and management of parathyroid disorders, differentiation of hypercalcemia etiologies, and clinical decision-making, particularly in patients with chronic kidney disease (CKD) [3]. Establishing RIs for iPTH is challenging, as iPTH concentrations are affected by clinical conditions and the composition of the reference population, underscoring the need for large cohorts of apparently healthy, VitD-replete individuals with normal renal function [4,5]. Consequently, published RIs differ depending on the applied inclusion criteria and the assays used, as current assays are not harmonized. It is also crucial to verify RIs provided by manufacturers to ensure the RI limits reflect the geographic patient population [6]. However, verification of the RIs using direct prospective studies can be difficult, as these studies require sample collection from healthy reference populations [7]. This challenge may be circumvented with indirect methods for RI estimation, which use statistical approaches to derive RIs from mixed populations of diseased and healthy individuals. These methods are more efficient and less labor intensive for the laboratory [8]. Some indirect RI estimation methods have been reported to tolerate a limited proportion of pathological results, commonly up to approximately 20%, and in some cases up to 30–40%, depending on the underlying distribution characteristics [9]. Nevertheless, it is recommended to substantially reduce the proportion of pathological results, for example, by considering the frequency of testing, the origin of the laboratory request, and/or accompanying laboratory results for each patient [10,11,12]. Therefore, in this case study on iPTH, and in light of the known challenges in establishing reliable RIs, like biological variation, preanalytical instability, and definition of healthy individuals, we aimed to minimize the proportion of pathological results through pre-processing as much as possible. As we hypothesized that this may influence the results, we used this setting to further evaluate which approaches are most effective for iPTH. To date, only a few studies have systematically addressed this issue for other analytes. Arzideh et al. (2021) investigated strategies for selecting among patients with multiple measurements [10], while Yang et al. (2023) [13] focused on outlier removal techniques and their combination with different data transformation methods or indirect approaches. However, beyond outlier handling, they did not examine combinations with other pre-processing strategies, as all datasets included only the first measurement per individual [13]. Although Ammer et al. (2023) provided step-by-step guidance for applying the refineR algorithm, which also includes recommendations for data pre-processing [14], and several review and opinion papers provide additional guidance, these approaches have not yet been systematically and comparatively evaluated [8,12,15,16,17]. Despite this lack of systematic evaluation, numerous studies have applied indirect methods while incorporating analyte-specific pre-processing strategies [18,19]. This development is highly valuable, as it demonstrates an increasing awareness of the importance of pre-processing in the application of indirect methods. However, the efficiency of some pre-processing strategies remains unclear.

Therefore, the present study aims to evaluate the impact of various pre-processing strategies to reduce the proportion of pathological samples in the dataset, in order to enable the derivation of reliable RIs for iPTH using three different indirect methods.

Measurements of iPTH obtained using the Abbott Alinity i analyzer from a single Norwegian center were analyzed. Following the application of various data pre-processing strategies, three indirect RI estimation methods representing distinct statistical approaches, RefLim, truncated maximum likelihood (TML), and refineR [20,21,22] were employed to derive the final RIs.

In addition, we assessed whether the resulting indirectly derived RIs are consistent with values reported in the literature. This approach was intended to determine whether indirect methods can be reliably applied for iPTH when routine laboratory data are appropriately pre-processed, as such methods represent an attractive alternative to direct approaches.

## 2. Materials and Methods

### 2.1. Dataset

All data analyzed were derived from routine laboratory reports using serum and lithium heparin plasma specimens. The data were collected over a period of four years (August 2019 to September 2023) at Stavanger University Hospital, Department of Medical Biochemistry, Norway which was accredited according to NS-EN ISO 15189 [23] at the time of data collection. Data included inpatient and outpatient data. Outpatient data made up more than 50% of the total dataset and iPTH requests were not restricted to clinical indications that would have a high probability of pathological iPTH results. The dataset included multiple measurements of the same patient (duplicates), where applicable.

### 2.2. Laboratory Tests

iPTH (non-STAT assay file) results were determined using the Abbott Alinity i immunoassay (Abbott GmbH, Wiesbaden, Germany) across multiple Alinity i analyzers with varying calibrator and reagent lots throughout the study period. Total calcium (Ca), total magnesium (Mg), alkaline phosphatase (ALP), enzymatic creatinine (used for estimated glomerular filtration rate-eGFR, using the CKD-EPI 2009 equation without race adjustment), and albumin (Alb: bromocresol purple) results were determined using Abbott ARCHITECT c16000 clinical chemistry assays (Abbott GmbH, Germany). The overall coefficient of variation (%CV) for iPTH ranged from 5% to 6%, based on internal quality control data collected from multiple Alinity i instruments during the study period. Ionized calcium (iCa) results were determined using ABL-800 (Radiometer, Krefeld, Germany). VitD was measured with LC-MS/MS, as described elsewhere [24].

### 2.3. Study Design

The study adopted a data-driven, sequential design, in which analyses were conducted stepwise, with earlier findings informing subsequent analyses, while also evaluating established findings reported in the literature.

Initially, the data were processed and cleaned to remove non-usable results, such as non-numerical values and records lacking essential meta-information (e.g., age or sex). This step is referred to as data cleaning and is defined here as the process of identifying, correcting, and removing erroneous data. It is distinguished from the subsequent step, data pre-processing. On the cleaned data, a drift analysis was conducted; visual inspection revealed no evidence of drift in iPTH results over the study period.

In this study, data pre-processing was defined as the pre-selection used to limit the number of subjects with disease included in a dataset. This definition was based on the work of Farrell and Nguyen (2019) [12]. In their study, potential pre-processing steps include the exclusion of extreme values, the exclusion of data from specific referral sites where patients are more likely to have significant disease, and the exclusion of repeated results [12]. In the present study, the impact of the first two approaches on indirectly derived RIs was investigated. In addition, another pre-processing step is evaluated: the exclusion of results based on accompanying laboratory test results for each patient. The exclusion of extreme values was applied to all datasets as a general pre-processing step; however, it is not considered part of the comparative analysis conducted in this study.

**Figure 1:**
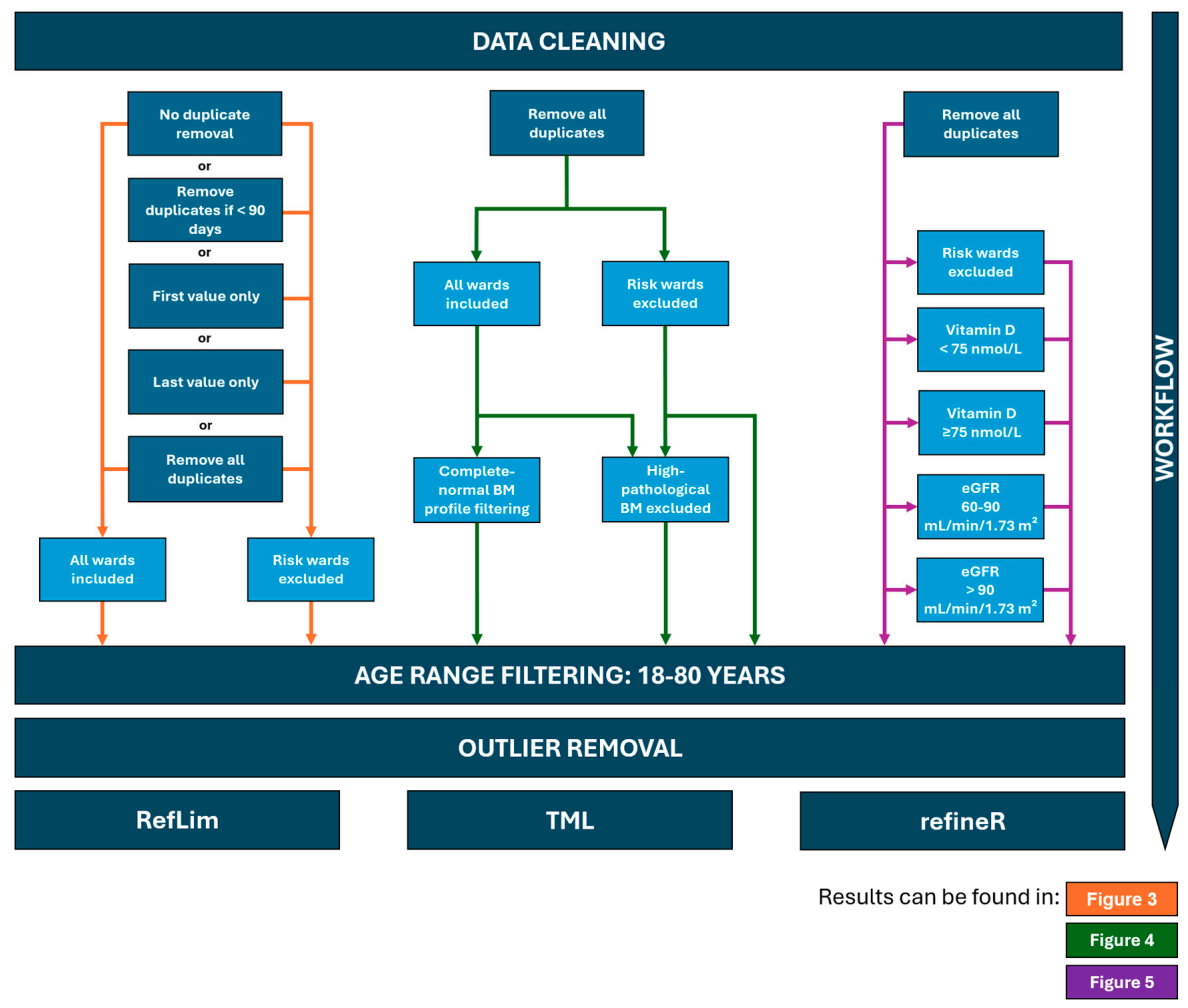
Graphical overview of the study data processing workflow. The figure illustrates the steps of three analyses conducted in this study for iPTH, each exploring different pre-processing or partitioning strategies. Color-coded arrows, representing the analysis pathways, indicate which figure in the Section 3 corresponds to each specific analysis path. BM = biomarker.

The main study analyses commenced:

1. **Assessment of Age- and Sex-Based Partitioning:** Following data cleaning, the potential need for partitioning iPTH RIs by age and sex was assessed. Selective data pre-processing steps and the indirect method RefLim (see below) were also applied to control for the dependence of the pathological fraction on age [25].
2. **Analysis of Data Pre-Processing—Risk Ward Exclusion, Duplicate Handling, and Additional Biomarker-Based Filtering and Their Impact on RI Estimation Using Indirect Methods:** We applied a set of different pre-processing approaches, subsequently estimated RIs using indirect methods (see below) and compared the results. A graphical overview of all analysis steps is provided in Figure 1. The analysis within this phase evaluated the effects of excluding data from risk wards, including intensive care and oncology units, where a high prevalence of pathological results is expected (Supplemental Table S1). Additionally, we investigated the influence of different duplicate-handling approaches, including retaining only the first measurement, retaining only the last measurement, and removing all patients with duplicate measurements to ensure statistical independence of the data [10]. For illustrative purposes, we compared these approaches to RI results generated from the dataset without duplicate removal, and to an approach excluding results occurring within 90 days of the preceding measurement. The underlying assumption was that a subsequent iPTH measurement obtained outside this time window would likely reflect a different clinical context (Figure 1, orange path).
  To assess whether the impact of the previous pre-processing steps could be further optimized, an additional biomarker-based filter was applied, excluding iPTH results if an abnormal value was simultaneously detected in a biomarker potentially influencing iPTH (Supplemental Table S2). Since not all biomarker results were available for all patients, we applied this filter at two different levels of stringency. First, we excluded iPTH results if one or more biomarker results were abnormal, regardless of whether all biomarkers had been measured. Second, we required that all biomarkers be available for a given patient and that all results be within the normal range. A graphical overview is also shown in Figure 1 (green path). The numerical RI results and corresponding confidence intervals for these two analyses and the subsequent two analyses are provided in Supplementary Table S3.
3. **VitD and eGFR Partitioning:** As iPTH levels decrease with increasing VitD concentrations [26,27,28] and an inverse association between eGFR and iPTH has been reported [29], the question arose as to whether pathological values of VitD and eGFR should not only be excluded during pre-processing as part of a broader biomarker-based filtering strategy, but whether these biomarkers, when considered individually, might themselves have an impact on the estimated RIs and potentially justify partitioning of iPTH RIs, depending on patients’ VitD and eGFR levels. The isolated effects of VitD and eGFR were therefore analyzed using datasets from which patients with duplicate measurements and data from risk wards had been excluded. A graphical overview is shown in Figure 1 (violet path).
4. **Capability of Indirect Methods to Separate the Pathological Fraction: Comparison with Conventional RI Estimation Methods and Extension to Albumin:** As a final question arising from the previous analyses, we investigated whether similar effects on calculated RIs could also be observed for another analyte when applying the same combinations of pre-processing steps and examined the extent to which indirect methods are capable of separating pathological results from the healthy distribution. Albumin was selected as a comparison analyte, as corresponding measurements were also available for large parts of the patients in the dataset. In addition to the indirect methods, two conventional RI estimation methods (parametric and nonparametric) were applied to the same pre-processing scenarios, reflecting different stages of pathological result reduction within the dataset. These approaches represent the standard methods used for RI determination in the context of direct sampling techniques [7]. As these methods do not inherently truncate the pathological fraction of the data, this comparison allowed us to assess the additional impact of indirect methods on the resulting RIs. Furthermore, this approach enabled the evaluation of potential differences between analytes with respect to how various pre-processing steps influence the calculated RIs.

Based on the results of step 1, all subsequent RI calculations were performed using the age cohort of 18 to 80 years and combining female and male subjects.

### 2.4. Indirect RI Estimation Methods

In this study, three indirect methods were used to estimate RIs: RefLim, truncated maximum likelihood (TML), and refineR [20,21,22]. RI calculations and corresponding 95% confidence intervals (CIs) were performed in R version 4.0.2 (R Core Team, 2024, Vienna, Austria, https://www.R-project.org/) using the packages reflimR (version 1.0.6), whose algorithm issues a warning when fewer than 200 observations remain after truncation [30], and refineR (version 1.6.2), for which results may become less reliable in datasets comprising fewer than 1000 observations [31]. The TML method, which requires at least 1000 observations [21], was implemented using an R function provided by the Deutsche Gesellschaft für Klinische Chemie und Laboratoriumsmedizin (DGKL). Where applicable, 200 bootstrap repetitions were used for the estimation of CIs.

### 2.5. Conventional RI Estimation Methods

For comparison to the indirect methods, standard parametric and nonparametric methods were used to calculate RIs. CIs for the RI limit point estimates were computed per standard procedures for parametric and nonparametric approach [32,33]. All calculations were performed in R Version 4.0.2. The numeric CI results can be found in Supplementary Table S3.

## 3. Results

### 3.1. Basic Data Cleaning and Analysis of iPTH Dependence on Age and Sex in the Study Dataset

**Figure 2:**
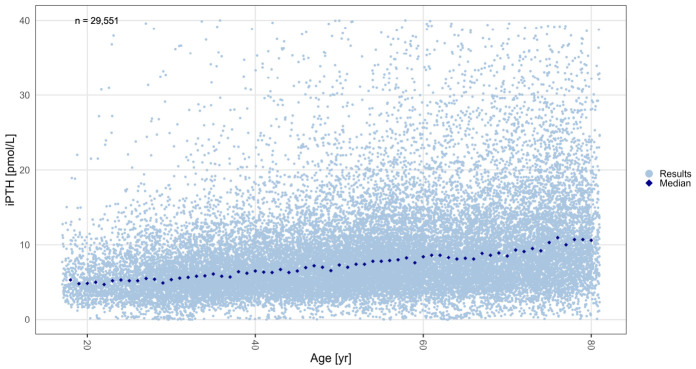
Scatterplot of iPTH results versus patient age (y-axis truncated). n = Total number of results after data cleaning.

A total of 33,854 iPTH results were obtained along with information on the age and sex of patients. After excluding patients not meeting the inclusion criteria or with missing data, 29,551 results for patients in the age range of 18 to 80 years remained. The median age of this cohort was 54 years, with 66.6% females and 33.4% males. This dataset included supposedly healthy and diseased patients. We observed that iPTH levels increased with age, with median values of about 5 pmol/L in the third decade and about 10 pmol/L in the ninth decade (Figure 2). The effect of age and sex was assessed by separate RI estimations. There were substantial overlaps between the confidence intervals and only minor differences between the point estimates of the upper refence limits (URLs) of different ages (Supplemental Figure S1) and sexes (Supplemental Figure S2). Therefore, age and sex were not considered in the remaining analyses.

### 3.2. Effect of Risk Ward Exclusion and Data Pre-Processing Based on Duplicate Handling (Number and/or Timing of Results) on RI Estimation Using Three Indirect Methods

**Figure 3:**
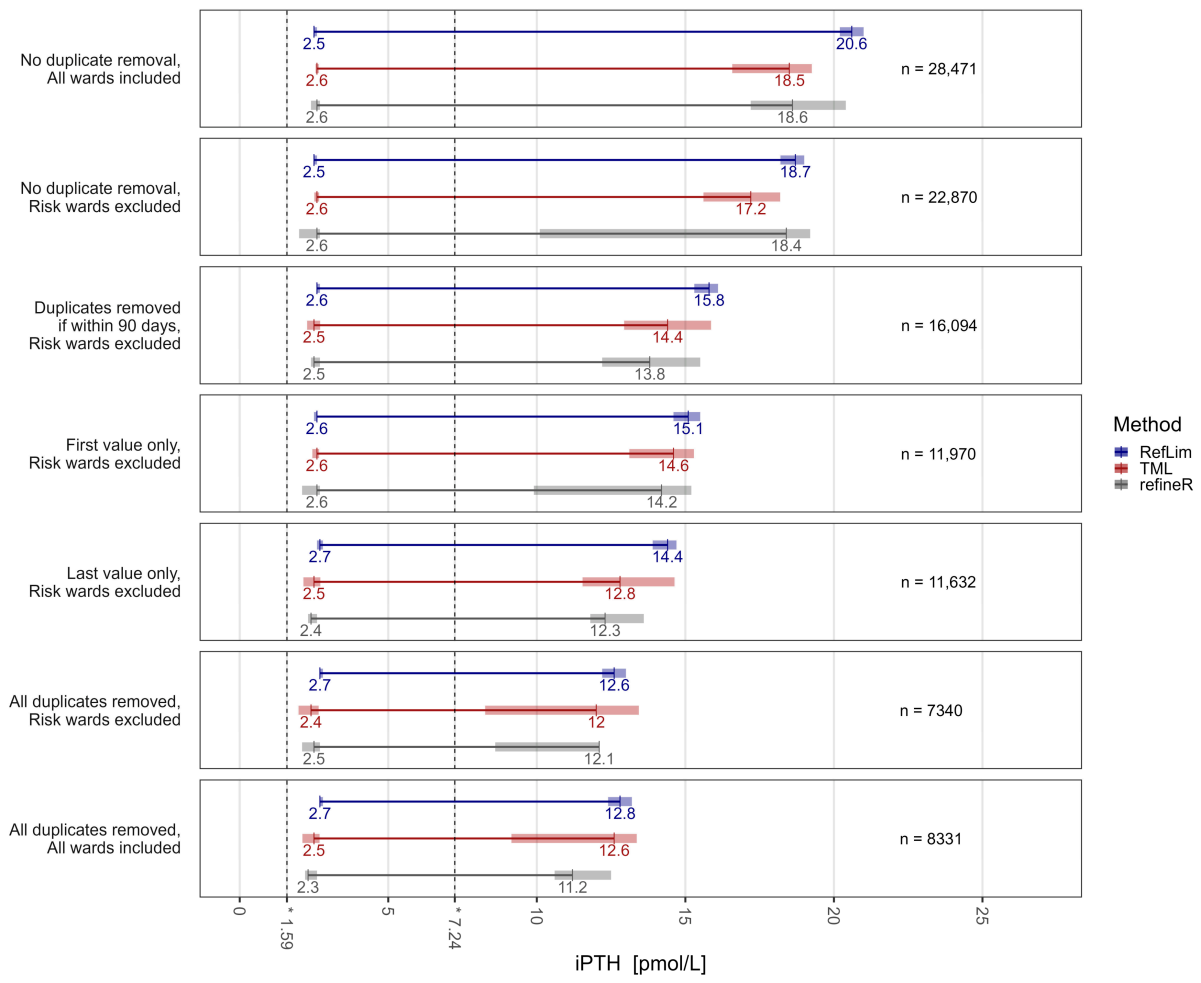
Influence of different duplicate removal options and the effect of risk wards exclusion on the estimated indirect RI for three independent indirect methods. Manufacturer-provided lower reference limits (LRL) and URL are indicated by vertical dashed lines and with asterisks (*). Calculated 95% CIs are shown as semi-transparent boxes. n denotes the number of reference individuals for the first value only, last value only, and all duplicates removed approaches. For the no duplicate removal and duplicates removed within 90 days approaches, n refers to the number of results included in the respective RI estimation.

To assess the effect of pathological data on the three indirect reference methods, RIs were calculated from different subsets of the data, as described below, and compared to the values provided in the manufacturers package insert (Figure 3). We analyzed the effect of risk wards (i.e., wards with presumable higher percentage of pathological iPTH results) removal. Using the dataset without the risk ward data, we investigated the influence of different duplicate removal methods, such as keeping only the first value, only the last value, excluding results occurring within 90 days of the preceding measurement, or removing all duplicates.

Since we compared three different methods across multiple scenarios, we focused primarily on overall trends in the point estimates while also considering the associated confidence intervals and the extent of their overlap. The numerical RI results and corresponding confidence intervals for this and all subsequent analyses are provided in Supplementary Table S3.

The removal of risk wards (Supplemental Table S1) had only a modest effect on the calculated RIs for both the less stringent (no duplicate removal) and most stringent (only patients with a single result) duplicate removal filters. In contrast, we found that lower reference limits (LRLs) were only slightly affected by the investigated duplicate filters, with values greater than or equal to 2.6 pmol/L. The URL estimates were significantly impacted by duplicate removals. The dataset, where only patients with a single available result were included (all patients with multiple results excluded), resulted in the lowest URL. When the analysis was restricted solely to the last available measurement, we observed only slightly higher URLs for refineR and TML, whereas the URL determined by RefLim was considerably higher. First-value-only filtering and excluding results occurring within 90 days of the preceding measurement resulted in significantly higher URLs. The highest URLs were observed in datasets where no exclusion criterion based on the number of results was applied. Compared to the unfiltered original dataset, the most stringent filter (singlicates—no risk wards) reduced the data size to only 24.8% of the starting size, while the remaining sample still met the minimum requirements of the applied indirect algorithms. Of note, the three different indirect methods resulted in comparable RIs in this setting, although CIs of TML and refineR method were wide and noticeably skewed towards lower values.

### 3.3. Additional Effect of Filtering by Biomarkers

**Figure 4:**
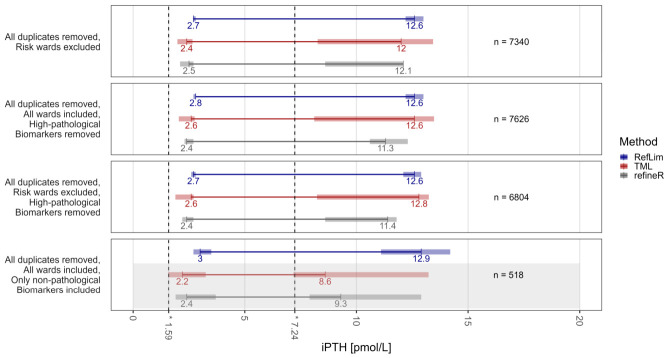
Influence of biomarker filtering on the determination of RIs by three indirect methods. For only non-pathological biomarkers, all biomarkers needed to be available and not in high pathological range. Gray shaded area: Recommended minimum sample size not met for RefineR and TML. Manufacturer-provided LRL and URL are indicated by vertical dashed lines and with asterisks (*). Calculated 95% CIs are shown as semi-transparent boxes. n denotes the number of individuals contributing to the respective RI estimation.

Patient data were filtered using site-specific exclusion criteria for pathological values (Supplemental Table S2) for iCa, Ca, Mg, ALP, albumin, VitD, and eGFR, as abnormalities in these parameters may influence iPTH levels. In the subset of patients with all duplicates removed, results were filtered by excluding iPTH values if one or more associated biomarkers were pathological (Figure 4). This filtering had no effect on the RIs determined by RefLim and TML and resulted in an insignificant and only slight decrease in URL using refineR. We further analyzed a dataset restricted to iPTH results with a complete set of non-pathological values across the entire biomarker panel. Following this additional filtering step, the number of available observations was substantially reduced and fell below the sample size considered suitable for the application of TML (minimum 1000 observations) [21] and refineR (approximately 1000 observations or more recommended) [31]. Consequently, RI estimation using these methods was not considered reliable for this dataset. In contrast, RefLim remained applicable (≥200 observations after truncation) [30], and the resulting RIs were comparable to those obtained under the other filtering conditions.

### 3.4. VitD and eGFR as Partitioning Factors

**Figure 5:**
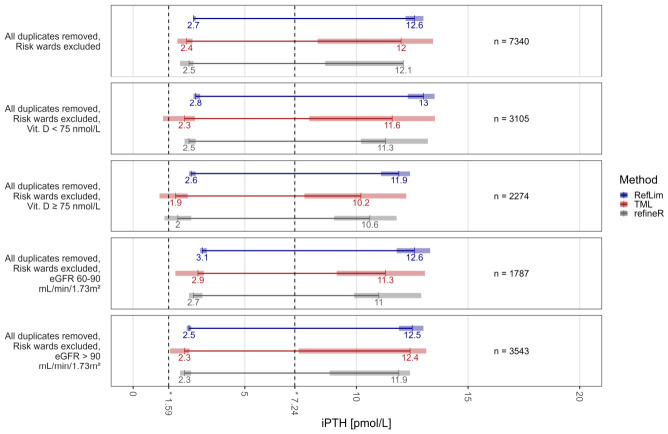
Partitioning according to VitD levels (<75 and ≥75 nmol/L) and eGFR (60 to 90 mL/min/1.73 m^2^ and >90 mL/min/1.73 m^2^) and estimation of indirect RIs of iPTH by three independent indirect methods. Manufacturer-provided LRL and URL are indicated by vertical dashed lines and with asterisks (*). Calculated 95% CIs are shown as semi-transparent boxes. n denotes the number of individuals contributing to the respective RI estimation.

VitD concentration and eGFR are important influencers of iPTH RIs. IPTH has been shown to decrease with increasing VitD levels, up to VitD values of approximately 75 to 100 nmol/L with the biggest effect till 50 nmol/L [26,27,28]. The dataset with all duplicates removed was evaluated for the effect of VitD levels on the iPTH RIs using a VitD cutoff of 75 nmol/L as partitioning criteria. For all three indirect methods, the URLs of the subgroup with VitD ≥ 75 nmol/L was lower compared to the subgroup < 75 nmol/L. The estimated URLs were higher when using RefLim compared to TML and refineR, respectively (Figure 5).

While the relationship between kidney function and the clearance of iPTH and its fragments is still under investigation, there is an inverse association between eGFR and iPTH [29]. Estimated URLs were comparable for patients with eGFR > 90 mL/min/1.73 m^2^ and eGFR 60–90 mL/min/1.73 m^2^ using the RefLim method. Using TML and refineR, we saw slightly higher URLs for the subgroup > 90 mL/min/1.73 m^2^ compared to the other eGFR subgroup (Figure 5).

### 3.5. Capability of Indirect Methods to Separate the Pathological Fraction: Comparison with Conventional RI Estimation Methods and Extension to Albumin

**Figure 6:**
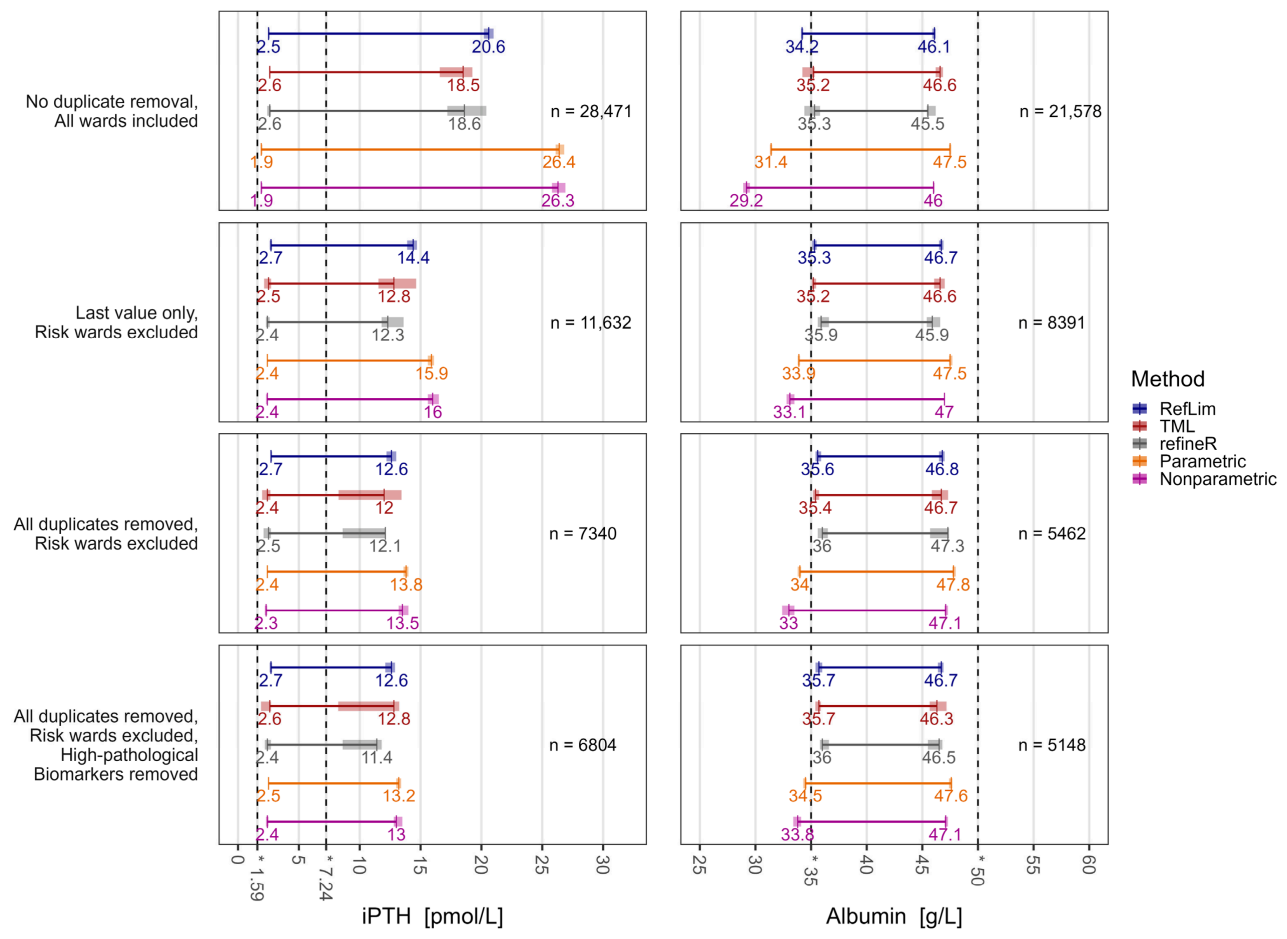
RIs estimated by indirect and conventional methods for iPTH and plasma albumin across pre-processing scenarios representing different degrees of pathological result reduction and varying proportions of assumed healthy individuals. Indirect methods were compared with conventional nonparametric and parametric approaches (parametric method applied after logarithmic transformation for iPTH) to evaluate the additional impact of pathological fraction truncation on RI estimation. The comparison between iPTH and albumin illustrates analyte-specific differences in how pre-processing strategies influence the calculated RIs. Manufacturer-provided LRL and URL are indicated by vertical dashed lines and with asterisks (*). Calculated 95% CIs are shown as semi-transparent boxes. n denotes the number of reference individuals for the last value only and all duplicates removed approaches. For the no duplicate removal approach, n refers to the number of results included in the respective RI estimation.

To estimate the capability of the indirect methods to separate pathological results and the reliability of the resulting RIs, we compared RIs derived from indirect methods with those obtained using conventional methods (parametric and nonparametric) for iPTH and albumin, both available in the same dataset (Figure 6).

As the conventional methods do not apply any additional model-based truncation of the pathological fraction, this comparison allowed us to evaluate the extent to which the method-specific truncation procedures of the indirect approaches further influenced the resulting RIs beyond the applied pre-processing steps. Differences between indirect and conventional estimates therefore provide insight into whether potentially pathological observations remain in the dataset after pre-processing and are subsequently excluded by the indirect methods.

Importantly, the primary purpose of this comparison was not to validate the indirect methods against conventional RI estimation. Rather, the inclusion of albumin enabled us to investigate whether the impact of pre-processing and subsequent indirect estimation was analyte-specific. Albumin exhibits a distribution that is well suited for indirect RI estimation, as the healthy population forms a dominant central distribution [34]. By comparing two analytes available within the same dataset, we were able to evaluate the ability of indirect methods to identify and separate the pathological fraction from the healthy population and to investigate whether this capability differs between analytes.

A convergence of RIs between the indirect and conventional methods for iPTH was observed with an overall decrease in URL with increasing stringency of applied filters. The conventional methods revealed higher values for the URL and lower values for the LRL for iPTH and albumin, respectively. For albumin, data pre-processing had only a small effect on the indirect methods; results of the indirect methods did not change with increasing stringency of applied filters. In addition, the LRLs obtained from indirect and conventional methods remained divergent even after all filters were applied (Figure 6, bottom panel).

The calculated CIs for iPTH showed a high variability between similar datasets (Figure 3) for TML and refineR with sometimes pronounced asymmetrical CIs and considerably wide ranges, whereas the CIs for RefLim was quite stable across all datasets and narrower than for the other two methods. In contrast, for albumin, the CIs were much narrower (Figure 6), even though the total numbers for the corresponding datasets were lower. The variability of the CIs between different similar datasets was, however, still observed for refineR.

## 4. Discussion

This study had two major objectives: to establish indirect RIs for iPTH on the Abbott Alinity i system using a single-center dataset by employing three methodically different indirect methods, but also to assess the need for and the efficiency of different data pre-processing strategies for removal of pathological results and their effect on the estimated RIs.

For our study, iPTH is an ideal marker, as on the one hand, there is a high need to accurately diagnose patients with CKD and mineral and bone disorders, and on the other hand, its highly skewed value distribution makes it well suited for exploring potential limitations of indirect algorithms in deriving reliable RIs. Current guidelines recommend monitoring of iPTH in relation to the URL, or multiples thereof. However, there is a great variability of URLs by different studies using mostly a direct approach, that could lead to differing clinical classifications of patients [4,5]. We used three indirect methods, with different statistical principles, to estimate RIs. Potentially observed discrepancies between the three methods might be an indicator of an ambiguous fitting of the healthy population [35,36].

A central observation in our study was that the iPTH RIs, especially the URL, were strongly dependent on data pre-processing steps, irrespective of the indirect method used. This effect was stronger than expected and implicates that all three indirect statistical algorithms were not capable of removing sufficiently the pathological data unless rigid data pre-processing was performed. We speculate that this might be due to an indistinct separation between healthy and diseased iPTH subjects and the high proportion of iPTH orders due to their use in monitoring diseased subjects. Strikingly, the strong dependency of the algorithms on data pre-processing was found even for our study cohort, which contained a high proportion of routine iPTH values (without clear clinical indication) of outpatient data, which is not typical for a usual hospital setting where most iPTH tests are performed in patients with CKD or severe bone disorders.

Notably, we observed that more rigorous duplicate removal methods led to increasingly lower URLs for indirect methods (Figure 3). This observation was largely independent of the indirect method employed, which reportedly has different robustness to the size of the pathological fraction [9]. These effects might be explained by an increasing removal of diseased (pathological) subjects starting with patients in ongoing treatment (duplicate iPTH testing within 90 days) which gradually decreases using the first value only (before treatment), the last value only (after treatment), up to the analysis of presumably healthy patients (single iPTH test in a four-year timeframe). Neither the additional removal of risk wards (Figure 3) nor the removal of data with pathological biomarkers (Figure 4) led to a substantial difference in the URL, indicating that pathological results were already sufficiently removed through the selection of patients with a single result. As the resulting RIs for the stringent filtering conditions are in line with recent direct RI studies (Table 1), our approach might be a more feasible and robust alternative for laboratories with sufficient data.

The duplicate removal pre-processing option is fast and easy to implement and worked well to remove pathological results before an assessment by indirect methods and does not require additional laboratory results, which are often not available and might lead to a strong reduction in the available data. Kalaria et al. used a preselection model for their indirect RI estimation based on clinical laboratory results (VitD, eGFR, Ca, Phosphate), leading to an overall RI of 2.0 to 11.2 pmol/L using the refineR algorithm [41]. In this study they included only patients with a VitD > 50 nmol/L. Similar to their results, our study demonstrated an RI of 2.2 to 10.8 pmol/L for subjects with a VitD ≥ 75 nmol/L using the refineR algorithm (Figure 5). In our study we saw no additional effect by removing patient data with other pathological biomarkers compared to the dataset with all duplicates removed, as the additional number of pathological results filtered out by biomarkers was low (Figure 4). The dataset, where results for all biomarkers were available and not highly pathological, was very small (n = 518) and below the requirement of TML and refineR, potentially limiting the real world applicability of this approach [9].

Overall, the RIs derived from the singlicate-only dataset compare well to the most other publications (see Table 1) with the exception of a few other studies, such as Cavalier et al., and the manufacturers instruction for use (IFU) [4,6].

We observed a considerably high variability of the calculated CIs for iPTH, especially for refineR, for very similar datasets, so the CIs should be interpreted with caution. We speculate that for heavily skewed distributions (like iPTH), the estimation for refineR and TML might be significantly influenced by small variations in the dataset, like the variations introduced by the bootstrapping algorithm used to calculate the CIs. However, CIs were more homogenous and stable for different datasets like albumin, which have a less skewed value distribution.

In contrast to Kalaria et al. and others we did not observe an increase in iPTH RIs with age (Supplemental Figure S1) [41,42,43]. In our dataset, excluding risk wards and removing all duplicates and applying the RefLim method, no clear age-related shift was evident, given the substantial overlap of confidence intervals across age groups. The median increase in iPTH values in the unfiltered dataset (Figure 2) and an observed small upwards trend of the URL point estimates for very high age groups (Supplemental Figure S1) were likely due to a higher prevalence of diseased subjects in older age groups. In contrast to studies that identified iPTH age-dependent RIs, we excluded patients based on frequency of iPTH testing rather than solely associated biomarkers. This difference in pre-processing might lead to the observed effects in addition to population differences. Our results indicate that consideration of the dependency of prevalence of diseased subjects on age is recommended before defining age-partitioning groups in real world datasets. Our study is corroborated by most direct studies that also did not report age-specific RIs (Table 1).

We also studied the effect of VitD for our dataset (Figure 5), as iPTH levels are dependent on the VitD status. The results of our subgroup analysis are in line with the RIs found by Deckers et al. and Minieri et al., even though Deckers et al. used different VitD levels for differentiation (50 vs. 75 nmol/L), see Table 1 [26,37].

Although kidney function has an effect on iPTH levels, we observed only minor differences between the analyzed subgroups, which might be attributed to the low accuracy of the eGFR estimation for mild and normal kidney function and thus largely overlapping calculated and potentially wrong CKD stages in our dataset [44]. A limitation of our study was the very low frequency of subjects with a more severely impaired kidney function. Thus, an analysis of these subgroups could not be performed.

Furthermore, limitations related to incomplete pre-analytical information should be acknowledged. Fasting status at the time of sample collection was not consistently documented across all observations. While it is assumed that the majority of measurements were obtained under morning fasting conditions, this cannot be verified for all cases. The subset of results with unknown or potentially non-fasting status is relatively small and is therefore expected to have only a minor impact on the overall findings, particularly if such observations were already excluded during data pre-processing. Nevertheless, the incomplete availability of fasting status information represents a limitation of this study and should be considered when interpreting the results.

The goal of data pre-processing is to sufficiently reduce the fraction of pathological results to enable the indirect methods to reliably characterize the non-pathological fraction. Theoretically, after performing sufficient filtering, the results of the indirect methods should not change significantly (Figure 3). The successful removal of pathological results through data filtering techniques was demonstrated by the convergence of the URLs obtained from indirect and conventional methods. (Figure 6). The URLs of the conventional methods were only slightly higher than those of the indirect methods for iPTH when multiple filtering combinations were used (all duplicates removed, risk wards excluded, high-pathological biomarkers excluded), indicating that the resulting population was close to an a posteriori-defined healthy population. Additionally, the comparison with the analysis of plasma albumin demonstrates that the strong dependance of the indirect methods on data pre-processing is not necessarily true for all biomarkers. For albumin, the results of the indirect methods changed little across different filtering conditions, indicating successful characterization of the non-pathological fraction by indirect methods. A possible explanation for this difference between different biomarkers might be the underlying result distribution, as also shown by Ammer et al. [9] and in our previous findings [35]. This underscores the importance of thoroughly evaluating analyte-specific data characteristics and implementing appropriate pre-processing strategies before applying indirect RI estimation methods. Reliance solely on the truncation procedures inherent to indirect methods may be insufficient, particularly for analytes with complex or highly skewed distributions and thereby bias the estimated RIs.

As derived new RIs are substantially higher than the manufacturer, their implementation would lead to a substantial reclassification of patients which should be discussed with the clinicians before changing the RI. However, the derived indirect RIs compare well to most recent publications using direct and indirect methods and should allow a better estimation of the iPTH status of the served population. As we had no clinical data and used only data from one site, it is advised to verify these RIs by each laboratory before implementation.

## 5. Conclusions

In conclusion, we demonstrate that biomarkers like iPTH require extensive data pre-processing steps to obtain reliable RIs using indirect methods. This requires a large starting data set, limiting the application of indirect methods for infrequently ordered tests. Even with a dataset containing four years of iPTH data, partitioning could only be done for some criteria, which might be even more challenging if the dataset contains more pathological data than ours. In addition, filtering by other biomarkers was only partially possible due to an insufficient number of patient data. Irrespective of these limitations, we were successful in estimating RIs for iPTH using indirect methods, which correspond well to published data. The parallel determination of RIs using conventional methods provides insight into the effectiveness of indirect methods in characterizing the non-pathological fraction of the data and the reliability of the resulting RIs. The analysis of plasma albumin showed that the indirect algorithms might be more efficient for other biomarkers, as shown before [45]. Laboratory professionals should be cautious to uncritically use indirect methods for RI estimation without careful analysis of the datasets. Laboratory professionals are advised to verify those results prior to implementation of an indirect method analysis. Our study highlights the value of comparing multiple methods and applying pre-processing techniques of varying stringency to contextualize the results calculated by the indirect methods.

## Figures and Tables

**Table 1:** RIs measured with Abbott Assay (pmol/L) in various published studies.

Publication	Sample Size (n)	25OH-VitD (nmol/L)	LRL (pmol/L)	URL (pmol/L)
Abbott instruction for use (IFU) [6]	143	not defined	1.59	7.24
	300	<50	2.8	13.0
Deckers [26] (LASA sample set) (2013)	438	≥50	2.7	11.1
	738	All	2.8	12.1
	183	<50	2.5	13.9
Deckers [26] (NESDA sample set) (2013)	450	≥50	2.0	9.4
	633	All	2.1	10.7
Minieri [37] (Blood Donors) (2021)	100	All	2.7	11.6
Minieri [37] (lab database) (2021)	495	All	2.0	11.9
	308	<75	1.6	13.5
Minieri [37] (by 25OH-VitD) (2021)	184	75–100	2.7	11.1
	85	>100	2.8	9.4
Andersen [38] (2023)	31	>50	2.4	10.9
Mirzazadeh [39] (nonparametric) (2023)	1727	50–200	3.0	13.7
Mirzazadeh [39] (parametric) (2023)	1727	50–200	2.9	14.1
La’ulu [40] (2010)	252	>50	1.9	8.5
Cavalier [4] (2012)	240	>75	1.7	6.9
Kalaria [41] (2024) (indirect)	5935	>50	2.0	11.2

## Data Availability

The datasets presented in this article are not publicly available due to data protection, as they contain sensitive patient data collected at the Department of Medical Biochemistry, Stavanger University Hospital, Stavanger, Norway.

## Supplementary Materials

The following supporting information can be downloaded at https://www.mdpi.com/article/10.3390/diagnostics16152305/s1, Figure S1: Age-Dependent RIs Derived Using RefLim in Overlapping 5-Year Intervals; Figure S2: Sex-Specific RIs for iPTH (18–80 Years) Using Three Indirect Methods; Table S1: List of Excluded Risk Senders; Table S2: Cutoffs Used for Exclusion of High-Pathological Biomarkers; Table S3: RI results and corresponding confidence intervals.

## Abbreviations

The following abbreviations are used in this manuscript:

VitD	Vitamin D
ALP	Alkaline phosphatase
BM	Biomarker
Ca	Calcium
CI	Confidence intervals
CKD	Chronic kidney disease
CV	Coefficient of variation
DGKL	Deutsche Gesellschaft für Klinische Chemie und Laboratoriumsmedizin
eGFR	Estimated glomerular filtration rate
iCa	Ionized calcium
IFU	Instruction for use
iPTH	Intact parathyroid hormone
LRL	Lower reference limit
Mg	Magnesium
PTH	Parathyroid hormone
RI	Reference interval
TML	Truncated maximum likelihood
URL	Upper reference limit
