# Supplementary material for: Intact Parathyroid Hormone Reference Intervals: Importance of Pre-Processing for Indirect Reference Interval Estimation
Source: Diagnostics (Basel). 2026 Jul 23;16(15):2305. doi: 10.3390/diagnostics16152305 (PMC13465239; doi:10.3390/diagnostics16152305)
Supplement: Supplementary file 1 [file diagnostics-16-02305-s001.zip › Table S1. List of Excluded Risk Senders.pdf]

| |
|---|
| Child |
| Child and youth |
| Dialysis |
| Endocrine surgery |
| Endocrinology |
| Error |
| Geriatry |
| Gynecology and Obstetrics |
| Hematology and oncology |
| ICU |
| Medical biochemisty |
| Nephrology |
| Neurology |
| Oncology and hematology |
| Obesity outpatient clinic |
| Palliative Care Unit |
| Pathology |
| Science |
| Urology |

Supplemental Table S1. List of Excluded Risk Senders
