# Supplementary material for: Intact Parathyroid Hormone Reference Intervals: Importance of Pre-Processing for Indirect Reference Interval Estimation
Source: Diagnostics (Basel). 2026 Jul 23;16(15):2305. doi: 10.3390/diagnostics16152305 (PMC13465239; doi:10.3390/diagnostics16152305)
Supplement: Supplementary file 1 [file diagnostics-16-02305-s001.zip › Table S2. Cutoffs Used for Exclusion of High-Pathological Biomarkers.pdf]

Ca (ionized)		Total Ca		eGFR	VitD		Total Mg		ALP	Albumin	
mmol/L		mmol/L		mL/min/1.73m^2	mmol/L		mmol/L		U/L	g/L	
<1.08	>1.45	<2.05	>2.65	<25	<25	>250	<0.61	>1.04	>500	<35	>50

Supplemental Table S2. Cutoffs Used for Exclusion of High-Pathological Biomarkers
