# Supplementary material for: Intact Parathyroid Hormone Reference Intervals: Importance of Pre-Processing for Indirect Reference Interval Estimation
Source: Diagnostics (Basel). 2026 Jul 23;16(15):2305. doi: 10.3390/diagnostics16152305 (PMC13465239; doi:10.3390/diagnostics16152305)
Supplement: Supplementary file 1 [file diagnostics-16-02305-s001.zip › Table S3. RI results and corresponding confidence intervals.pdf]

Analyte	Pre-processing used	N	Method for RI estimation	LRL	CI (LRL)	URL	CI (URL)
iPTH [pmol/L]	All duplicates removed, Risk wards excluded	7340	RefLim	2.7	(2.7 / 2.8)	12.6	(12.2 / 13)
iPTH [pmol/L]	All duplicates removed, Risk wards excluded	7340	TML	2.4	(2 / 2.7)	12	(8.3 / 13.4)
iPTH [pmol/L]	All duplicates removed, Risk wards excluded	7340	refineR	2.5	(2.1 / 2.7)	12.1	(8.6 / 12.1)
iPTH [pmol/L]	First value only, Risk wards excluded	11970	RefLim	2.6	(2.5 / 2.6)	15.1	(14.6 / 15.5)
iPTH [pmol/L]	First value only, Risk wards excluded	11970	TML	2.6	(2.4 / 2.7)	14.6	(13.1 / 15.3)
iPTH [pmol/L]	First value only, Risk wards excluded	11970	refineR	2.6	(2.1 / 2.7)	14.2	(9.9 / 15.2)
iPTH [pmol/L]	Last value only, Risk wards excluded	11632	RefLim	2.7	(2.6 / 2.8)	14.4	(13.9 / 14.7)
iPTH [pmol/L]	Last value only, Risk wards excluded	11632	TML	2.5	(2.1 / 2.7)	12.8	(11.5 / 14.6)
iPTH [pmol/L]	Last value only, Risk wards excluded	11632	refineR	2.4	(2.3 / 2.6)	12.3	(11.8 / 13.6)
iPTH [pmol/L]	All duplicates removed, All wards included, High-pathological Biomarkers removed	7626	RefLim	2.8	(2.7 / 2.8)	12.6	(12.2 / 13)
iPTH [pmol/L]	All duplicates removed, All wards included, High-pathological Biomarkers removed	7626	TML	2.6	(2 / 2.8)	12.6	(8.1 / 13.5)

iPTH [pmol/L]	All duplicates removed, All wards included, High-pathological Biomarkers removed	7626	refineR	2.4	(2.3 / 2.7)	11.3	(10.6 / 12.3)
iPTH [pmol/L]	All duplicates removed, All wards included	8331	RefLim	2.7	(2.7 / 2.8)	12.8	(12.4 / 13.2)
iPTH [pmol/L]	All duplicates removed, All wards included	8331	TML	2.5	(2.1 / 2.7)	12.6	(9.1 / 13.4)
iPTH [pmol/L]	All duplicates removed, All wards included	8331	refineR	2.3	(2.2 / 2.6)	11.2	(10.6 / 12.5)
iPTH [pmol/L]	Duplicates removed if within 90 days, Risk wards excluded	16094	RefLim	2.6	(2.6 / 2.7)	15.8	(15.3 / 16.1)
iPTH [pmol/L]	Duplicates removed if within 90 days, Risk wards excluded	16094	TML	2.5	(2.3 / 2.7)	14.4	(12.9 / 15.9)
iPTH [pmol/L]	Duplicates removed if within 90 days, Risk wards excluded	16094	refineR	2.5	(2.4 / 2.7)	13.8	(12.2 / 15.5)
iPTH [pmol/L]	No duplicate removal, Risk wards excluded	22870	RefLim	2.5	(2.5 / 2.6)	18.7	(18.2 / 19)
iPTH [pmol/L]	No duplicate removal, Risk wards excluded	22870	TML	2.6	(2.5 / 2.7)	17.2	(15.6 / 18.2)
iPTH [pmol/L]	No duplicate removal,	22870	refineR	2.6	(2 / 2.7)	18.4	(10.1 / 19.2)

	Risk wards excluded						
iPTH [pmol/L]	No duplicate removal, All wards included	28471	RefLim	2.5	(2.5 / 2.6)	20.6	(20.2 / 21)
iPTH [pmol/L]	No duplicate removal, All wards included	28471	TML	2.6	(2.6 / 2.7)	18.5	(16.6 / 19.3)
iPTH [pmol/L]	No duplicate removal, All wards included	28471	refineR	2.6	(2.4 / 2.7)	18.6	(17.2 / 20.4)
iPTH [pmol/L]	All duplicates removed, Risk wards excluded, eGFR 60-90 $mL/min/1.73m^2$	1787	RefLim	3.1	(3 / 3.3)	12.6	(11.8 / 13.3)
iPTH [pmol/L]	All duplicates removed, Risk wards excluded, eGFR 60-90 $mL/min/1.73m^2$	1787	TML	2.9	(1.9 / 3.2)	11.3	(9.1 / 13.1)
iPTH [pmol/L]	All duplicates removed, Risk wards excluded, eGFR 60-90 $mL/min/1.73m^2$	1787	refineR	2.7	(2.5 / 3.1)	11	(9.9 / 12.9)
iPTH [pmol/L]	All duplicates removed, Risk wards excluded, eGFR > 90 $mL/min/1.73m^2$	3543	RefLim	2.5	(2.4 / 2.6)	12.5	(11.9 / 13)
iPTH [pmol/L]	All duplicates removed, Risk wards excluded, eGFR > 90 $mL/min/1.73m^2$	3543	TML	2.3	(1.7 / 2.5)	12.4	(7.4 / 13.1)
iPTH [pmol/L]	All duplicates removed, Risk wards excluded,	3543	refineR	2.3	(2.1 / 2.6)	11.9	(8.8 / 12.4)

	eGFR > 90 mL/min/1.73m^2						
iPTH [pmol/L]	All duplicates removed, Risk wards excluded, High-pathological Biomarkers removed	6804	RefLim	2.7	(2.6 / 2.8)	12.6	(12.1 / 12.9)
iPTH [pmol/L]	All duplicates removed, Risk wards excluded, High-pathological Biomarkers removed	6804	TML	2.6	(1.9 / 2.7)	12.8	(8.2 / 13.2)
iPTH [pmol/L]	All duplicates removed, Risk wards excluded, High-pathological Biomarkers removed	6804	refineR	2.4	(2.2 / 2.7)	11.4	(8.6 / 11.8)
iPTH [pmol/L]	All duplicates removed, Risk wards excluded, Vit. D ≥ 75 nmol/L	2274	RefLim	2.6	(2.5 / 2.8)	11.9	(11.1 / 12.4)
iPTH [pmol/L]	All duplicates removed, Risk wards excluded, Vit. D ≥ 75 nmol/L	2274	TML	1.9	(1.2 / 2.4)	10.2	(7.7 / 12.2)
iPTH [pmol/L]	All duplicates removed, Risk wards excluded, Vit. D ≥ 75 nmol/L	2274	refineR	2	(1.4 / 2.6)	10.6	(9 / 11.8)
iPTH [pmol/L]	All duplicates removed, Risk wards excluded,	3105	RefLim	2.8	(2.7 / 3)	13	(12.3 / 13.5)

	Vit. D < 75 nmol/L						
iPTH [pmol/L]	All duplicates removed, Risk wards excluded, Vit. D < 75 nmol/L	3105	TML	2.3	(1.3 / 2.8)	11.6	(7.9 / 13.5)
iPTH [pmol/L]	All duplicates removed, Risk wards excluded, Vit. D < 75 nmol/L	3105	refineR	2.5	(2.3 / 2.8)	11.3	(10.2 / 13.2)
iPTH [pmol/L]	All duplicates removed, All wards included, Only non-nathological Biomarkers included	518	RefLim	3	(2.7 / 3.5)	12.9	(11.1 / 14.2)
iPTH [pmol/L]	All duplicates removed, All wards included, Only non-nathological Biomarkers included	518	TML	2.2	(1.6 / 3.3)	8.6	(7.2 / 13.2)
iPTH [pmol/L]	All duplicates removed, All wards included, Only non-nathological Biomarkers included	518	refineR	2.4	(1.9 / 3.7)	9.3	(7.9 / 12.9)
Albumin [g/L]	All duplicates removed, Risk wards excluded, High-pathological Biomarkers removed	5148	RefLim	35.7	(35.4 / 36)	46.7	(46.4 / 46.9)
Albumin [g/L]	All duplicates removed,	5148	TML	35.7	(35.4 / 35.9)	46.3	(45.7 / 47.2)

	Risk wards excluded, High-pathological Biomarkers removed						
Albumin [g/L]	All duplicates removed, Risk wards excluded, High-pathological Biomarkers removed	5148	refineR	36	(35.8 / 36.6)	46.5	(45.5 / 46.8)
Albumin [g/L]	All duplicates removed, Risk wards excluded	5462	RefLim	35.6	(35.4 / 35.9)	46.8	(46.5 / 47)
Albumin [g/L]	All duplicates removed, Risk wards excluded	5462	TML	35.4	(35.2 / 35.7)	46.7	(45.9 / 47.3)
Albumin [g/L]	All duplicates removed, Risk wards excluded	5462	refineR	36	(35.6 / 36.5)	47.3	(45.7 / 47.4)
Albumin [g/L]	Last value only, Risk wards excluded	8391	RefLim	35.3	(35.1 / 35.5)	46.7	(46.5 / 46.9)
Albumin [g/L]	Last value only, Risk wards excluded	8391	TML	35.2	(35.1 / 35.4)	46.6	(46.1 / 47)
Albumin [g/L]	Last value only, Risk wards excluded	8391	refineR	35.9	(35.6 / 36.6)	45.9	(45.4 / 46.6)
Albumin [g/L]	No duplicate removal, All wards included	21578	RefLim	34.2	(34.1 / 34.3)	46.1	(45.9 / 46.2)
Albumin [g/L]	No duplicate removal, All wards included	21578	TML	35.2	(34.2 / 35.3)	46.6	(46.2 / 46.9)
Albumin [g/L]	No duplicate removal, All wards included	21578	refineR	35.3	(34.4 / 35.8)	45.5	(45.4 / 46.2)

iPTH [pmol/L]	No duplicate removal, All wards included	28471	Nonparametric method	1.9	(1.9 / 2)	26.3	(25.8 / 26.9)
iPTH [pmol/L]	No duplicate removal, All wards included	28471	Parametric method (log)	1.9	(1.9 / 2)	26.4	(26.1 / 26.8)
Albumin [g/L]	All duplicates removed, Risk wards excluded	5462	Nonparametric method	33	(32.4 / 33.5)	47.1	(47 / 47.3)
Albumin [g/L]	All duplicates removed, Risk wards excluded	5462	Parametric method	34	(33.8 / 34.1)	47.8	(47.7 / 48)
Albumin [g/L]	All duplicates removed, Risk wards excluded, High-pathological Biomarkers removed	5148	Nonparametric method	33.8	(33.4 / 34.1)	47.1	(47 / 47.3)
Albumin [g/L]	All duplicates removed, Risk wards excluded, High-pathological Biomarkers removed	5148	Parametric method	34.5	(34.3 / 34.6)	47.6	(47.4 / 47.7)
Albumin [g/L]	Last value only, Risk wards excluded	8391	Nonparametric method	33.1	(32.8 / 33.5)	47	(47 / 47)
Albumin [g/L]	Last value only, Risk wards excluded	8391	Parametric method	33.9	(33.8 / 34)	47.5	(47.4 / 47.7)
Albumin [g/L]	No duplicate removal, All wards included	21578	Nonparametric method	29.2	(28.9 / 29.5)	46	(46 / 46.1)
Albumin [g/L]	No duplicate removal, All wards included	21578	Parametric method	31.4	(31.3 / 31.5)	47.5	(47.4 / 47.6)
iPTH [pmol/L]	All duplicates removed,	6804	Nonparametric method	2.4	(2.3 / 2.5)	13	(12.8 / 13.5)

	Risk wards excluded, High-pathological Biomarkers removed						
iPTH [pmol/L]	All duplicates removed, Risk wards excluded, High-pathological Biomarkers removed	6804	Parametric method (log)	2.5	(2.5 / 2.5)	13.2	(13 / 13.4)
iPTH [pmol/L]	All duplicates removed, Risk wards excluded	7340	Nonparametric method	2.3	(2.2 / 2.4)	13.5	(13.2 / 14)
iPTH [pmol/L]	All duplicates removed, Risk wards excluded	7340	Parametric method (log)	2.4	(2.4 / 2.4)	13.8	(13.6 / 14)
iPTH [pmol/L]	Last value only, Risk wards excluded	11632	Nonparametric method	2.4	(2.3 / 2.4)	16	(15.6 / 16.5)
iPTH [pmol/L]	Last value only, Risk wards excluded	11632	Parametric method (log)	2.4	(2.4 / 2.4)	15.9	(15.6 / 16.1)

N denotes the number of reference individuals for the first value only, last value only, and all duplicates removed approaches. For the no duplicate removal and duplicates removed within 90 days approaches, N refers to the number of results included in the respective RI estimation.
